# Calcium oscillations in mesenchymal stem cells, a control on cell cycle progression to influence cell fate towards proliferation or differentiation?

**DOI:** 10.1186/s13287-025-04454-8

**Published:** 2025-08-21

**Authors:** Leslie A. Vallet, Marina Sánchez-Petidier, Romain Fernandes, Nataliia Naumova, Caterina Merla, Claudia Consales, Giorgia Innamorati, Franck M. André, Lluis M. Mir

**Affiliations:** 1 Metabolic and Systemic Aspects of Oncogenesis for New Therapeutic Approaches (METSY), UMR 9018, CNRS, Gustave Roussy, Université Paris-Saclay, PR2, 114 Rue Edouard Vaillant, 94805 Villejuif, France; 2 Neural Circuits and Behaviour Lab, Fundación Hospital Nacional de Parapléjicos, Castilla La Mancha Health Research Institute (IDISCAM), 45071 Toledo, Spain; 3 Division of Biotechnologies, Italian National Agency for New Technologies, Energy and Sustainable Economic Development (ENEA), 00123 Rome, Italy

**Keywords:** Cell proliferation, Electrical stimulation, Cell cycle control, Microsecond pulsed electric fields, Electroporation, Electropermeabilization, Calcium electroporation, Calcium oscillations frequency, Adipogenic differentiation, Osteogenic differentiation

## Abstract

**Background:**

Under regular culture conditions, mesenchymal stem cells (MSCs) exhibit cytosolic calcium concentration oscillations (Ca^2+^ oscillations), that change, especially in frequency, after the differentiation of the MSCs. Ca^2+^ oscillations are known to encode important information in frequency and amplitude, ultimately controlling many cellular processes such as proliferation and differentiation. Previous studies evidenced that decreasing the frequency of Ca^2 +^ oscillations by physical means can facilitate osteodifferentiation of MSCs. Understanding the relationships between Ca^2 +^ oscillations and MSCs proliferation or differentiation appears necessary in the attractive perspective of influencing cell fate by controlling Ca^2 +^ signaling.

**Methods:**

Using fluorescence microscopy we evaluated the evolution of Ca^2+^ oscillations throughout the adipogenic and osteogenic differentiation processes. Then, using electrical stimulation with microsecond pulsed electric fields (µsPEFs), we manipulated the frequency of Ca^2+^ oscillations in MSCs and measured its consequences on cell growth.

**Results:**

Although the evolution of the Ca^2 +^ oscillation frequencies differed between the adipogenic and osteogenic differentiation pathways in early stages of differentiation, we observed common features in the late stages: a progressive decrease in the Ca^2 +^ oscillations frequencies, before their complete arrest as the differentiations reached their term. It has been reported that most cells undergoing differentiation experience a concomitant commitment to terminal differentiation and cell cycle exit, and prior to this, lengthened G1 phases, where the molecular competition between mitogenic and differentiating signals occurs. A relationship between the frequency of Ca^2+^ oscillations and the progression of the cell cycle, through some Ca^2 +^ sensitive molecular factors, could explain the evolutions of the frequencies of Ca^2+^ oscillations observed during proliferation and differentiation. We hypothesized that increasing the frequency of Ca^2+^ oscillations would promote proliferation, while decreasing it would promote differentiation under differentiating conditions. Using electrical stimulation with µsPEFs, we manipulated the frequency of Ca^2+^ oscillations in MSCs and its increase actually promoted cell proliferation.

**Conclusions:**

Manipulating the frequency of Ca^2 +^ oscillations influences the cell fate of MSCs. We propose hypotheses on the actors that could link the Ca^2 +^ oscillation frequencies with proliferation and differentiation processes, based on data available in the literature.

## Background

Since their discovery by Friedenstein and colleagues in the bone marrow [1–4], mesenchymal stem cells (MSCs) have received a growing interest from the scientific community for the purpose of regenerative medicine, in cell-based [5, 6], and even cell-free therapies, beneficiating from the properties of their secretome [7]. This major interest raised not only due to the wide multipotency of the MSCs, but also from other roles they fulfill in the organism, related to their immunomodulatory properties [8], their antioxidant properties [9], or also the rescuing effect they can have towards damaged cells, performing mitochondrial transfer for instance [10]. In another respect, it has been evidenced that MSCs exhibit Ca^2+^ oscillations (oscillatory variations in their cytosolic Ca^2 +^ concentrations in time) in regular culture conditions, during the G1 and S phases of the cell cycle [11]. Such oscillations are known to depend on the mobilization of Ca^2+^ from intracellular stores through the IP3 receptors and to depend also on store-operated calcium entry to sustain the oscillations [12]. It was further demonstrated that extracellular ATP contributes to the generation of these Ca^2+^ oscillations via an autocrine/paracrine signaling through P2Y1 receptors, activating PLC-β, which subsequently generates IP3, triggering the mobilization of Ca^2+^ from intracellular stores [13].

Ca^2+^ is a universal cellular second messenger, the signal being mainly encoded through repetitive oscillations known to carry important information in their frequency, amplitude and shape. This information is subsequently decoded by proteins whose activities are Ca^2+^ -sensitive and which are involved in many cellular processes such as proliferation and differentiation [14, 15]. It has also been shown that these Ca^2+^ oscillations are modified in differentiated MSCs cells, especially in their frequencies (e.g., from 4 to 5 oscillations visualized in 10 min in undifferentiated human bone marrow derived MSCs, down to 1 oscillation visualized in 10 min for MSCs induced in osteogenic differentiation for 3 weeks) [16]. Understanding the relationships between Ca^2+^ oscillations and MSCs proliferation or differentiation is necessary for the attractive perspective of influencing the cell fate of MSCs by controlling the Ca^2+^ signaling. Previous reports have shown that direct current (DC) stimulation, which decreased the frequency of Ca^2+^ oscillations in MSCs undergoing osteogenic differentiation, had an impact on the differentiation process [17]. A common feature shared by (and not limited to) the osteogenic and adipogenic differentiation pathways, is the associated commitment to terminal differentiation and cell cycle exit [18, 19]. Prior to these events, it is well established for the adipogenic differentiation model [18], and more broadly described as a general mechanism [20], that cells require lengthened G1 phases, where the molecular competition between mitogenic and differentiating signals occurs, to get a chance to commit to terminal differentiation and exit from the cell cycle. In this study, we first aimed at characterizing the evolution of Ca^2 +^ oscillations during osteogenic and adipogenic differentiation processes in comparison to Ca^2+^ oscillations observed under classical conditions of cell proliferation. Interestingly, we observed a progressive decrease in Ca^2 +^ oscillation frequencies followed by a complete arrest in both osteogenic and adipogenic late stages of differentiation until cells commitment to terminal differentiation. This led us to focus on the common events experienced by cells in the late stages of differentiation. We drew on the existing literature to connect the dots between our observations on the Ca^2+^ oscillation frequency patterns in proliferation and differentiation, the molecular aspects of the coordination between cell proliferation and differentiation [18, 20], and the regulation of actors involved in cell cycle progression through Ca^2+^ oscillation in a frequency-specific manner [21]. The information from the literature reported here above was then used to develop strategies aimed at controlling cell fate by modulating Ca^2+^ oscillations using a class of specific electrical signals known as microsecond pulsed electric fields (µsPEFs), which have previously been shown to provide a very flexible tool for controlling Ca^2+^ oscillations in MSCs [22]. In the experiments here reported, the stimulations with µsPEFs are based on those described in [22] but the µsPEFs parameters are optimized to achieve a long-term control of Ca^2+^ oscillations through repeated µsPEFs applications.

## Methods

### Cell culture and differentiation

Human adipose-derived MSCs were isolated from surgical waste of individuals undergoing elective lipoaspiration. Samples were obtained after written informed consent from all of the donors, in accordance with French and European legislations. The lipoaspirates were surgical waste and, as such, the French legislation (Art.L. 1245–2 du Code de la Santé Publique) establishes that authorization from an ethics committee is not required. The isolation procedure of the cells as well as their characterization by surface antigen analysis and assessment of multipotency as preconized in [23] were already described in [24]. Cells were grown in DMEM high glucose with GlutaMAX (Gibco, 31,966–047) supplemented with 10% fetal bovine serum (Sigma, F7524), 100 U/mL penicillin and 100 mg/mL streptomycin (Gibco, 15,140,122), at 37 °C in a humidified incubator with 5% CO_2_. Cells passage was done once or twice a week. Differentiations were induced at passages comprised between 7 and 9. Prior to differentiation, cells were seeded at a density of 10,000 cells/cm^2^ and left in culture for 3–4 days to reach confluency. After reaching full confluency, the normal culture medium was removed and replaced with differentiation medium (this being considered the first day of differentiation, D0). The osteogenic differentiation medium was composed of complete MEM α high glucose with GlutaMAX (Gibco, 32,561,037) supplemented with 100 nM of dexamethasone (Sigma, D2915), 200 μM of ascorbic acid (Sigma, 49,752) and 10 mM of glycerol 2-phosphate (Sigma, 50,020). The osteogenic differentiation lasted 4 weeks (during which the medium was changed twice a week). The adipogenic differentiation medium was composed of complete DMEM high glucose with GlutaMAX supplemented with 1 μM dexamethasone, 200 μM indomethacin (Sigma, I7378), 500 μM 3-isobutyl-1-methylxantine (Sigma, I5879) and 10 μg/mL insulin (Sigma, I9278). The adipogenic differentiation lasted 3 weeks (during which the medium was changed 3 times a week).

### Assessment of differentiation

#### Alkaline phosphatase assay

According to the stage of differentiation, cells were harvested using trypLE (Gibco, 12,604,013) (week 1 of differentiation) or trypLE and collagenase I (Gibco, 17,018,029) (4 mg/mL) (weeks 2 to 4). A pre-treatment with EDTA (10 mM in PBS) was necessary to remove calcium deposits at week 4. In order to test the ALP activity (osteogenic differentiation), harvested cells were centrifuged at 300 × g for 5 min and resuspended in the assay buffer (Abcam, ab83369) following a ratio of 300,000 cells for 600 μl of assay buffer. The resuspended samples were centrifuged at 13,000 × g for 15 min at 4 °C. The supernatant was transferred to a new tube. Then 80 μl of the supernatant were pipetted in each of four wells of a 96-well plate. Two of these wells served to determine the background through the simultaneous addition of 50 μl p-nitrophenyl phosphate (pNPP), 5 mM (Abcam, ab83369) and 20 μl of stop solution (Abcam, ab83369). For the two remaining wells, 50 μl of pNPP 5 mM were added. After 60 min at 25 °C, 20 μl of the stop solution were added to the wells and the plate was read in a spectrophotometer (TECAN, Lyon, France) to measure the optical density at 405 nm. The quantity of p-nitrophenol in each well was determined using a standard curve established using pNPP in known quantities and purified ALP enzyme (Abcam, ab83369).

### Alizarin red staining

Osteogenic cells were labelled with alizarin red staining according to the following protocol: cells were washed in PBS and fixed in 95% methanol for 10 min. Alizarin red S (Sigma, A5533) solution 2% in deionized water (pH adjusted to 4.1–4.3 with 10% ammonium hydroxide) was added and incubated for 5 min, then rinsed multiple times with water until no eluting stain could be seen and cells were imaged under an epifluorescence microscope to visualize the calcium deposits (Zeiss Axiovert 100 with a Zeiss AxioCam Hrc camera controlled by Axio Vision 4.6 software; Carl Zeiss, Rueil-Malmaison, France).

### Bodipy staining

Adipogenic cells were stained with bodipy (Sigma, 790,389), as follows: the cell medium was removed, and the cells were incubated for 30 min with a fresh medium containing 10 µM of bodipy. Incubation medium was removed and replaced with fresh medium. Cells were imaged under the epifluorescence microscope (Zeiss Observer with a Zeiss AxioCam Hrc camera controlled by Zen software; Carl Zeiss) with a FITC filter set (BP_ex_ = 475/35 nm–BP_em_ = 513–556 nm) under constant conditions of time exposure, lamp power, lenses and temperature.

### Oil red O staining

Prior to staining, cells were washed in PBS (Gibco, 10,010,023) and fixed in 10% neutral buffered formalin (Sigma, F8775) for 20 min at room temperature. The staining solution was prepared combining three volumes of Oil Red O (Sigma, O0625) stock solution at 5 g/L in isopropanol with two volumes of deionized water. Fixed cells were washed twice with deionized water and incubated with isopropanol/H2O (60/40) for 5 min at room temperature. Cells were then incubated with the staining solution for 20 min at room temperature. Cells were subsequently washed with deionized water until no eluting staining could be observed. Stained lipid vesicles were observed under the Zeiss light microscope.

### Calcium oscillations visualization and analysis

In order to visualize calcium oscillations at different stages of cell differentiation, cells were loaded with 5 µM Fluo-4-AM (Life technologies, F14217/λ_ex_ = 494 nm, λ_em_ = 506 nm) and 0.2 µg/mL Hoechst 33,342 (λ_ex_ = 361 nm, λ_em_ = 497 nm) for 30 min at 37 °C, 5% CO_2_. In order to avoid any bias due to the introduction of fresh medium, part of the medium used for the incubation with Fluo-4-AM/ Hoechst 33,342 was the medium in which the cells were in culture at this moment. After incubation, cells were washed twice with warm PBS and put back in culture medium (in the remaining fraction of previous culture medium, which had been set aside beforehand). Cells were then imaged by fluorescence microscopy (Zeiss Observer with a Zeiss AxioCam Hrc camera controlled by Zen software; Carl Zeiss) for 15 min in a temperature/CO_2_ controlled chamber ensuring 37 °C/5% CO_2_ for the whole time of acquisition. The acquisition parameters were set to record Hoechst fluorescence with DAPI filter set (BPex: 377/60 nm/BPem: 447/60 nm) and Fluo-4 fluorescence with FITC filter set. The acquisition consisted in one single image of Hoechst fluorescence at the beginning followed by time-lapse imaging recording of the Fluo-4 fluorescence every 10 s for 15 min (500 ms exposure with HPX 120 C, Leistungselektronik JENA GmbH fluorescence excitation lamp). The obtained images were treated with CellProfiler 2.0 to delimit the nucleus of each cell and to extract the variations of Fluo-4 fluorescence in time for each cell, with the delimited nucleus defining the area of analysis. These data were subsequently analyzed with a customized version of the Matlab program *Spectral Analysis of Calcium Oscillations by Per Ulhen* [25], adapted to perform analysis on large number of cells, but keeping the same spectral analysis based on Fourier transform than the original program. This analysis allowed us to extract for each cell the principal frequency (the frequency for which the power spectral density is the highest) of the oscillations of the Ca^2+^ signal. Three independent experiments were conducted for each differentiation process, at several weeks intervals, with similar results. Indeed, identical trends were observed. However, since cells were not synchronized at the beginning of the differentiation induction, some variability in the individual cell evolution kinetics was observed within each experiment, also reflected between the repeats of the experiments. Final analysis was thus performed using all the cells in a single data set. At the beginning of the differentiation processes, between 100 and 150 cells were analyzed per experimental point and per experiment. Therefore, since each differentiation was repeated three times, the minimal number of cells analyzed per condition was 397. Since some proliferation occurred during the first steps of the differentiation processes, particularly during the osteogenic differentiation, the maximal number of cells analyzed per experimental point and experiment reached 763 to 935 cells (leading to a total of 2566 analyzed cells) in the osteogenic differentiation, and 183 to 345 cells (leading to a total of 750 analyzed cells) in the adipogenic differentiation. Statistics were performed with non-parametric one-way ANOVA (Kruskal–Wallis test by ranks, based on the median value of Ca^2+^ oscillations frequencies) and Dunn’s multiple comparisons post-hoc test for each condition compared to the control condition MSCs ctrl (day 0). It must be noted that in Fig. 2 (and in Fig. 6), Ca^2+^ oscillations frequencies at around 2 or 3 mHz (corresponding to an oscillation every 300 + to 500 + seconds) were not considered as real Ca^2+^ oscillations, but as artifacts due to the global trend of fluorescence in the acquisition (modulated for instance by phenomena such as probe photobleaching) and the subsequent processing of the data with the Matlab program involving a step of trend correction. The evaluation of the trend component was performed by approximating the Ca^2+^ signal recorded to a polynomial function, whose degree is selected depending on the original shape of the signal. However, the polynomial function could by itself show oscillatory behavior and could hence perturb the spectral analysis [25]. Cells which did exhibit Ca^2+^ oscillations were not affected by this artifact in their principal frequencies, but this was the case of cells which did not display Ca^2+^ oscillations.

### Electrical stimulation and proliferation analysis

24 h before stimulation, MSCs were seeded at a density of 3 × 10^3^ cells/cm^2^ in a defined area of 2cm^2^. Cells were exposed in their classical culture medium to 30 bipolar 25 + 25 µsPEFs at 300 V/cm applied at a frequency of 17 mHz (1 pulse per minute, for a duration of 30 min, once a day). The cells were either exposed 1, 2, 3, or 4 days to this stimulation. The exposure system for this electrical stimulation has been described in [26]. Under the different conditions assessed, the proliferation was evaluated by staining the cells with Hoechst 33,342, imaging the whole well in a Cytation 1 (Agilent BioTek), and proceeding to the counting of all the nuclei in the whole well. The results are shown as the fold change in cell number as compared to day 1.

## Results

### Characterization of MSCs osteogenic differentiation

**Fig. 1:**
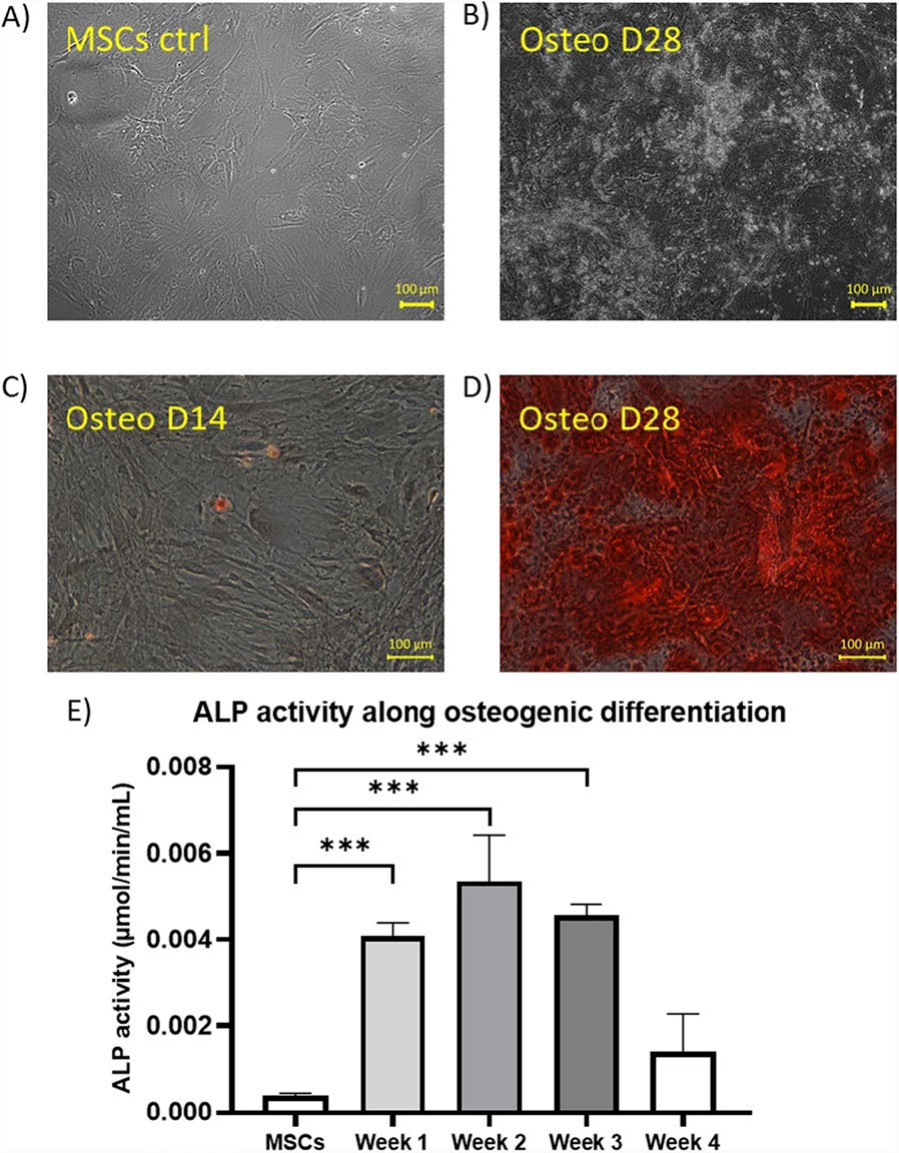
Characterization of MSCs differentiation: Morphological aspect (visualized with light microscopy) of **A** the undifferentiated control MSCs (day 0) and **B** of the MSCs differentiated in the osteogenic lineage at day 28 of differentiation. Alizarin Red staining (visualized with light microscopy) of MSCs differentiated in the osteogenic lineage **C** at day 14 of differentiation with the formation of the first calcium deposits and **D** at day 28 of differentiation with the presence of an important amount of calcium deposits. **E** Alkaline phosphatase (ALP) activity along the osteogenic differentiation of MSCs—data are presented as mean ± SD and are the result of 3 biologically independent replicates (at the exception of week 4 which is the result of 2 biologically independent replicates). Statistics were performed using one-way ANOVA with Dunnett’s multiple comparisons test ***p < 0.001

We followed osteogenic differentiation through cell morphological changes (Fig. 1B) compared to undifferentiated MSCs (day 0) (Fig. 1A). We performed the alizarin red staining of the Ca^2+^ deposits to confirm the mineralization of the extracellular matrix (ECM). We observed that these deposits started to appear by the second week of differentiation (Fig. 1C) and accumulated during the course of the differentiation until day 28 (Fig. 1D). We also characterized the osteogenic differentiation of MSCs by monitoring the alkaline phosphatase activity (ALP) along the differentiation (Fig. 1E). We observed that the ALP activity increased from the first week of differentiation and reached its maximum at the second week of differentiation, before decreasing again (Fig. 1E).

### Frequency of calcium oscillations along osteogenic differentiation

**Fig. 2:**
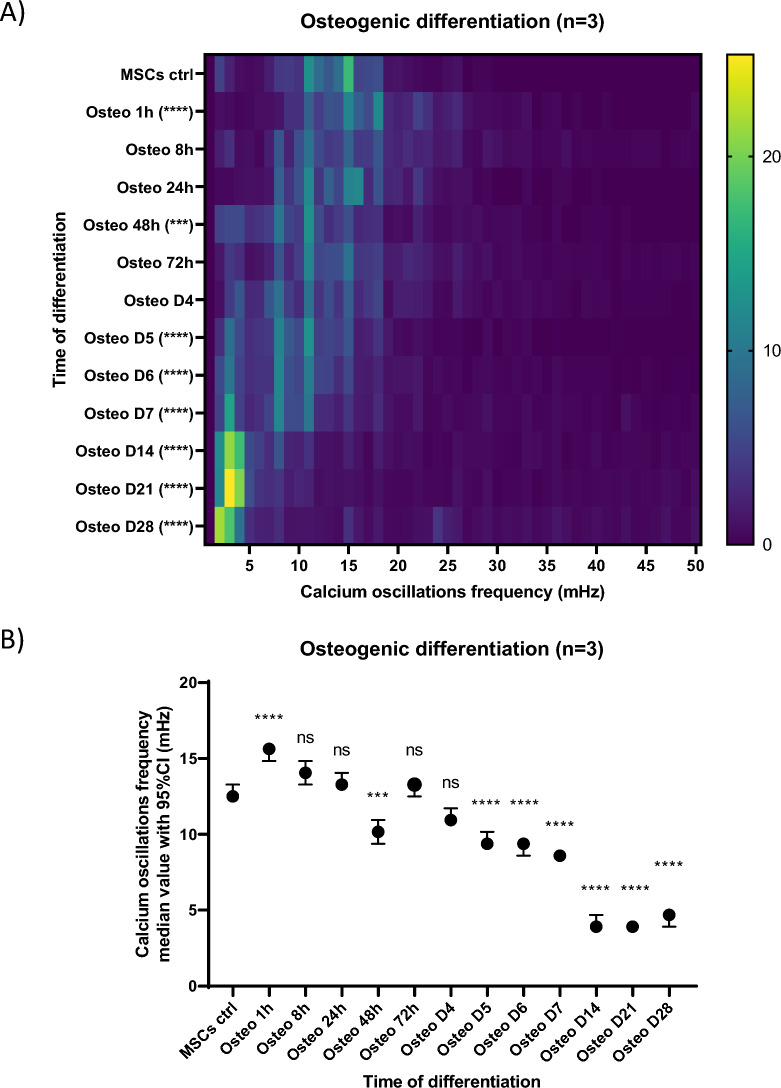
Evolution of the frequency of calcium oscillations along the osteogenic differentiation of MSCs. **A** Frequency distribution of calcium oscillations frequencies observed at various timepoints of the osteogenic differentiation. For a given timepoint condition (y-axis; note that the timescale is not continuous), the percentage of cells exhibiting given frequencies of Ca^2+^ oscillations (x-axis, in mHz) is indicated by a color code whose legend is at the right of the heatmap. The number of cells analyzed in one condition could vary from 397 (for MSCs ctrl) up to 2566 (for osteo D28). **B** Median value with 95% confidence interval of calcium oscillations frequencies observed at various timepoints of the osteogenic differentiation. Data are shown as compiled results of experiment triplicates (n = 3), each experiment evaluating calcium oscillations in hundreds of cells per timepoint condition. Statistics were performed with non-parametric one-way ANOVA (Kruskal–Wallis test by ranks) and Dunn’s multiple comparisons post-hoc test for each condition compared to the control condition MSCs ctrl. "ns” not significant, *p* < 0.01**, *p* < 0.001***, *p* < 0.0001****

Aiming at analyzing the evolution of the Ca^2+^ oscillations frequencies all along the osteogenic differentiation, we observed that, in the hour following the induction of this differentiation the frequency of the Ca^2+^ oscillations exhibited a slight increase as compared to undifferentiated MSCs in classical culture conditions (day 0) (Figs. 2A and B). Indeed, 1 h after the induction of differentiation, 52% of cells exhibited oscillations above 15 mHz, against only 23% in undifferentiated MSCs (Fig. 2A). In undifferentiated MSCs usually 54% of cells exhibit Ca^2+^ oscillations frequencies comprised between 10 and 15 mHz. We also found that 7% of cells do not display Ca^2+^ oscillations (Fig. 2A), consistent with the fact that Ca^2+^ oscillations have been previously reported to occur only in the G1 and S phases of the cell cycle in MSCs [11]. Ca^2+^ oscillations then resumed to frequencies similar to those observed in undifferentiated MSCs during the first days of the osteogenic differentiation (until day 4) and then tended to decrease as the differentiation proceeded (from day 5) (Figs. 2A and B). Finally, from day 14 on, the Ca^2+^ oscillations progressively stopped in an increasing number of cells until the differentiation arrived to its term (Fig. 2A) [25].

**Fig. 3:**
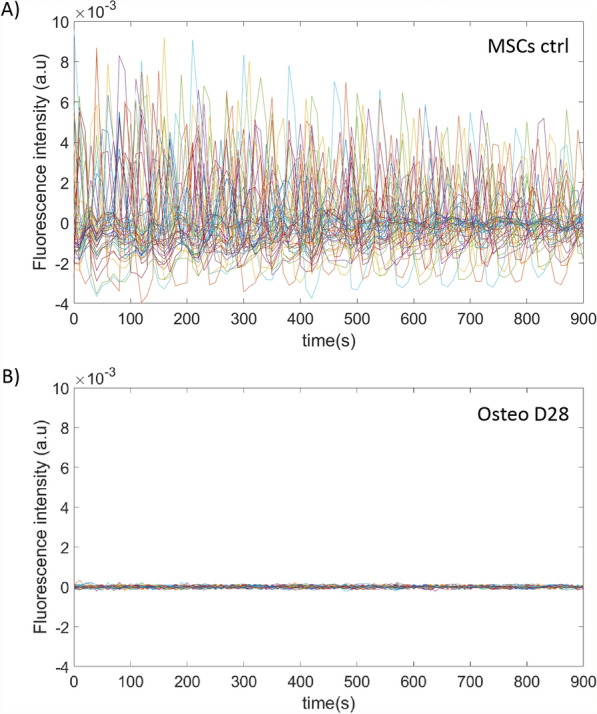
Examples of calcium signals in time (represented by fluorescence intensity of Fluo-4 in arbitrary unit (a.u)). Panel **A** for undifferentiated control MSCs (day 0). Panel **B** for cells in osteogenic differentiation for 28 days. In each panel, the trace of 50 individual cells are represented in different colors

Fluo-4 AM sensitively detects Ca^2+^ concentrations from 100 to 1 µM [27] and has one of the highest dynamic ranges among synthetic Ca^2+^ dyes [28]. It is therefore suitable for the analysis of Ca^2+^ events such as Ca^2+^ oscillations, where the Ca^2+^ concentration varies greatly over time. However, it is not a ratiometric fluorescent dye and as such, is not suitable for absolute quantitative analysis of the amplitude of Ca^2+^ oscillations. Nevertheless, its use clearly sustained that the observation of the Ca^2+^ relative levels evolution over time was consistent with the presence of Ca^2+^ oscillations in undifferentiated control MSCs (day 0) (Fig. 3A) and their absence in cells differentiated in the osteogenic lineage (Fig. 3B).

### Characterization of adipogenic differentiation

**Fig. 4:**
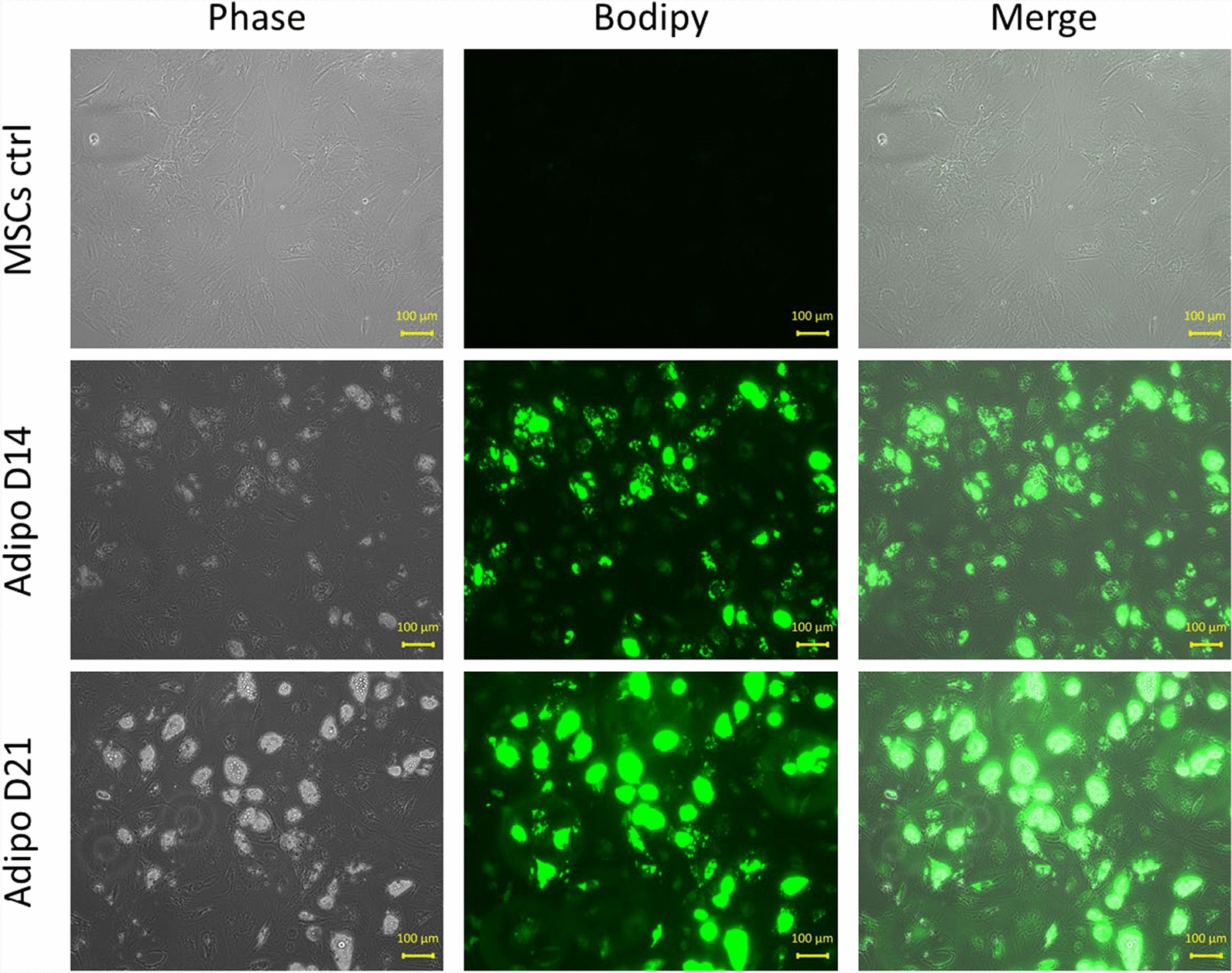
Characterization of MSCs differentiation. Left column: Morphological aspect (under phase contrast microscopy) of the undifferentiated control MSCs (upper row) and of MSCs differentiated in the adipogenic lineage at day 14 of differentiation (middle row) and at day 21 of differentiation (lower row). Middle column: Bodipy staining of control MSCs and of MSCs differentiated in the adipogenic lineage at day 14 and 21 of differentiation. Right column: merged images of the left and middle columns showing the evolution of the differentiation in time. Images representative of three independent experiments

**Fig. 5:**
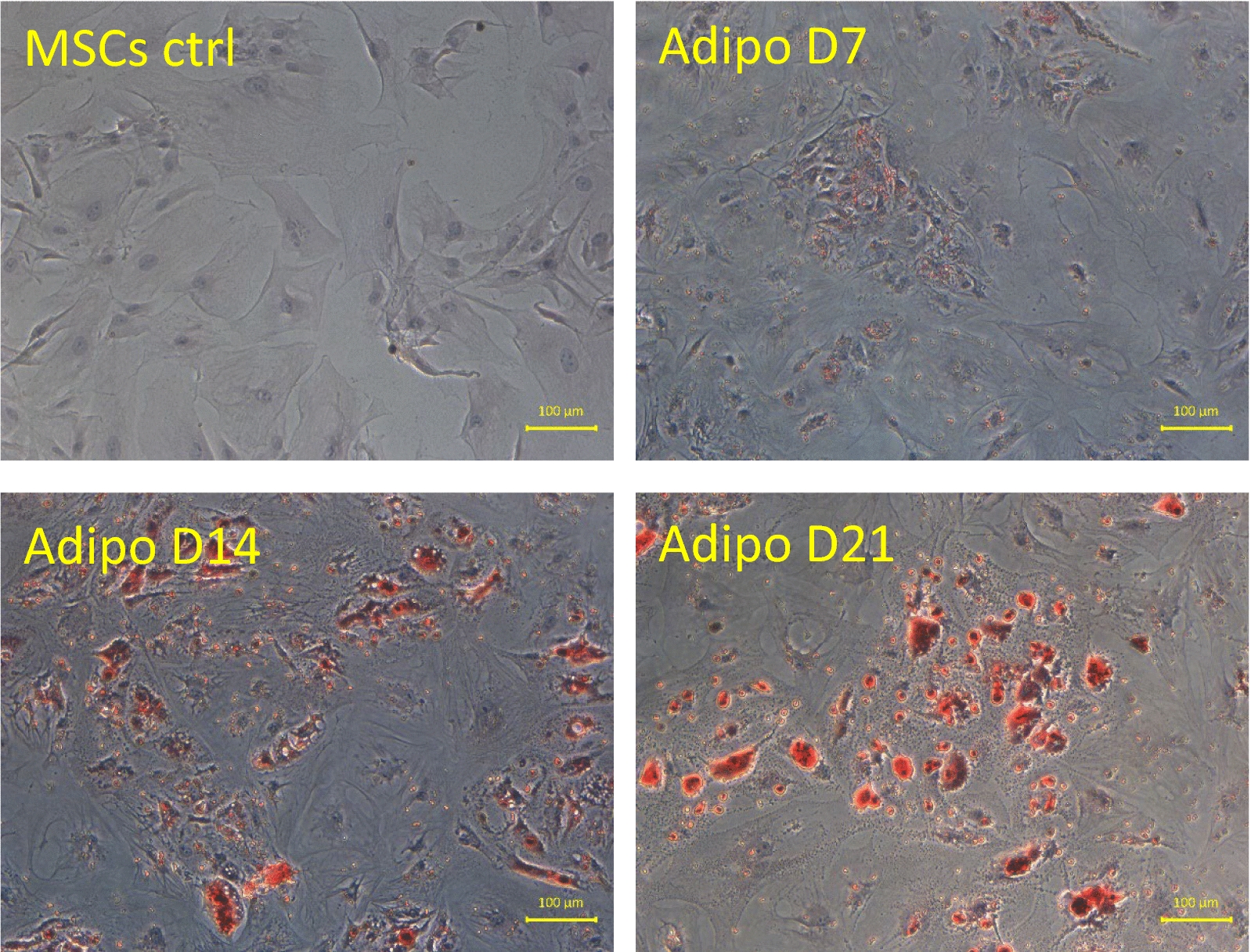
Characterization of adipogenic MSCs differentiation. Microscopy images of the progressive accumulation of lipids in the differentiating cells labelled with Oil red O

The adipogenic differentiation was followed through the cell morphological changes (Fig. 4, left column) and was characterized by the Bodipy staining (Fig. 4, middle column). The merged images of the phase contrast microscopy and of the bodipy staining of the same region is shown in Fig. 4, (right column). Adipogenic differentiation was also characterized by the Oil Red O staining (Fig. 5) of intracellular lipid vesicles. The first lipid vesicles could be observed around day 7 of differentiation (Fig. 5) and were progressively visible in more and more cells, with the number of lipid vesicles as well as their size increasing with the time of differentiation (Figs. 4 and 5).

### Frequency of calcium oscillations along adipogenic differentiation

**Fig. 6:**
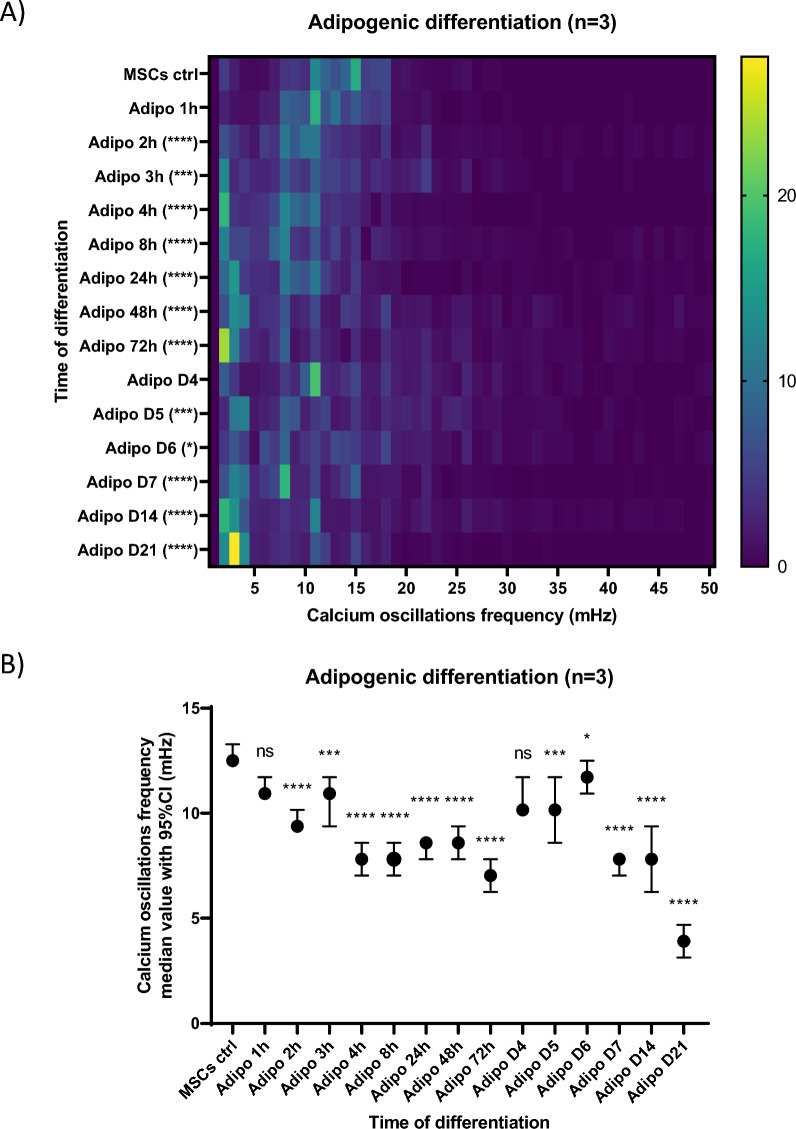
Evolution of the frequency of calcium oscillations during the adipogenic differentiation of MSCs. **A** Frequency distribution of the calcium oscillations frequencies observed at various timepoints of the adipogenic differentiation. For a given timepoint (y-axis; note that the timescale is not continuous), the percentage of cells exhibiting indicated frequencies of Ca^2+^ oscillations (x-axis, in mHz) is indicated by a color code whose legend is at the right of the heatmap. The number of cells analyzed in one condition varied from 397 cells (for MSCs ctrl) up to 750 cells (for adipo D21). **B** Median value with 95% confidence interval of calcium oscillations frequencies observed at various timepoints of the adipogenic differentiation. Data are shown as compiled results of experiment triplicates (n = 3), each experiment evaluating calcium oscillations in hundreds of cells per timepoint condition. Statistics were performed with non parametric one-way ANOVA (Kruskal–Wallis test by ranks) and Dunn’s multiple comparisons post-hoc test for each condition compared to the control condition MSCs ctrl. "Ns not significant, *p* < 0.01**, *p* < 0.001***, *p* < 0.0001****

In contrast to the osteogenic differentiation, upon the induction of adipogenic differentiation, a decrease in the frequency of the Ca^2+^ oscillations was observed from the first day of differentiation, even within the first hours (Figs. 6A and B). Indeed, from 2 h after the induction of adipogenic differentiation, less than 55% of cells exhibited Ca^2 +^ oscillations frequencies above 10 mHz as compared to 77% for undifferentiated MSCs (day 0) (Fig. 6A). The global tendency for reduced Ca^2+^ oscillation frequencies, with distinct populations of cells exhibiting different frequencies (Fig. 6), remained quite similar over a time period of almost 7 days. There were two exceptions (the adipo day 4 and adipo day 6 conditions): both corresponded to conditions assessed one day after the change for fresh differentiation medium, with fresh fetal bovine serum (FBS) containing mitogenic factors. The mitogenic factors contained in the FBS activate TKRs and PLC downstream, directly contributing to IP3 production, evidenced to be involved in the first mobilization of Ca^2+^ from the ER in the mechanism of Ca^2+^ oscillations in MSCs [12]. At day 14 of adipogenic differentiation 31% of the cells did no longer exhibit Ca^2+^ oscillations, and the proportion of these cells increased until the end of the differentiation (40% of cells not exhibiting Ca^2+^ oscillation). More specifically, in the context of the adipogenic differentiation, differentiated cells could be easily distinguished by their characteristic morphology, containing lipid vesicles, as well as a nucleus that is smaller compared to that of the undifferentiated MSCs [29]. In the course of the differentiation, all the cells presenting the morphological features of mature adipocytes displayed no oscillation, while the cells in which Ca^2+^ oscillations were still observed were the ones with morphological features of undifferentiated MSCs (data not shown). In other words, adipogenic differentiation appears to be very asynchronous, rapidly displaying the presence of two main populations: the cells already differentiated (expressing recognizable adipocytes morphological characteristics) that do not oscillate, and the cells still undifferentiated that continue to display Ca^2+^ oscillations. This is consistent with previous reports that adipocytes do not exhibit spontaneous Ca^2+^ oscillations [13, 30].

### Increasing the Ca^2+^ oscillations frequency of MSCs is associated with cell proliferation:

Thus, in adipogenic differentiation, a relationship seems to exist between the persistence of the Ca^2+^ oscillations and the maintenance of the undifferentiated character. Moreover, in osteogenic differentiation, Ca^2+^ oscillations frequencies increased in the hour following the induction of the differentiation (Figs. 2A and B) and then the oscillations persisted at frequencies similar to those of undifferentiated MSCs during the first days of the differentiation process (until day 4) (Figs. 2A and B) at a time when we previously had shown that cells still actively divide [31]. Several other arguments from the literature, reported in the discussion section, support a relationship between sustained Ca^2+^ oscillations in MSCs and cell proliferation, and provide insights into frequencies of Ca^2+^ oscillations that might selectively modulate the activity of actors involved in the cell cycle progression. For example, the RAS small GTPase was most strongly activated at 17 mHz in a study comparing Ca^2+^ oscillation frequencies between 3 and 17 mHz [21]. In the case of the spontaneous oscillation patterns of MSCs under classical culture conditions, we observed different populations of cells oscillating at different frequencies, with the majority of cells (54%) having Ca^2+^ oscillation frequencies comprised between 10 and 15 mHz (Figs. 2 and 6). Taken together, our results and the knowledge available in the literature led us to hypothesize that increasing the Ca^2+^ oscillation frequencies compared to those observed in undifferentiated MSCs under regular culture conditions could promote faster cell proliferation, whereas decreasing it, in differentiating conditions (i.e. in presence of differentiating factors) would promote faster differentiation. To directly test the hypothesis that increasing the Ca^2+^ oscillation frequency could increase cell proliferation, we applied µsPEFs. This tool has previously been shown to add Ca^2+^ oscillations similar to natural ones when used at low electric field amplitudes [22]. Aiming at increasing the frequency of Ca^2+^ oscillations compared to the spontaneous ones, the stimulation simply consists in applying these µsPEFs at the desired frequency, with each pulse generating a Ca^2+^ oscillation similar to the spontaneous ones. In this study, we used bipolar 25 + 25 µsPEFs at 300 V/cm to perform this stimulation.

**Fig. 7:**
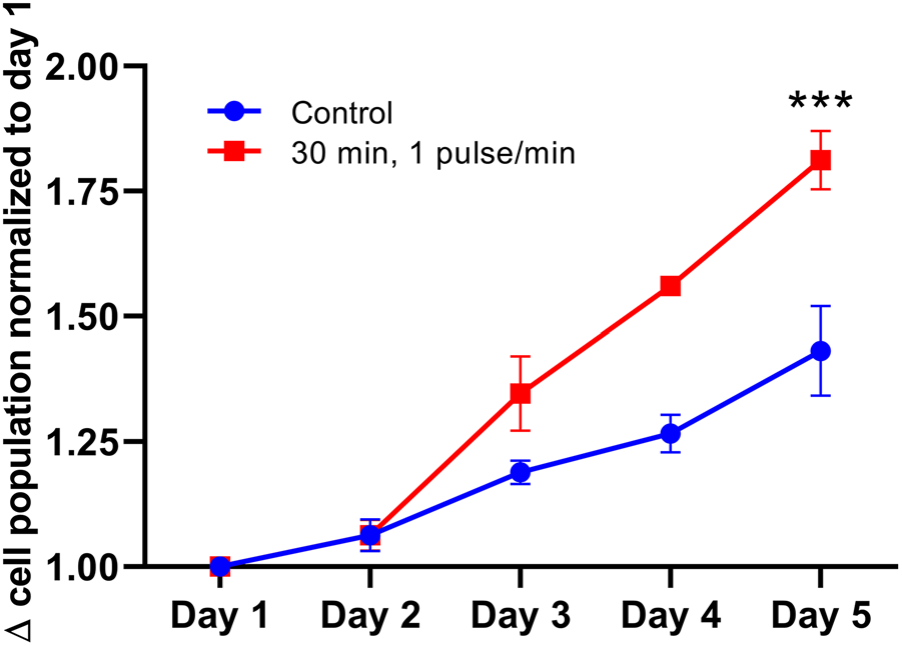
Fold change in cell number as compared to day 1, in control conditions (blue curve, round dots) and stimulated (1 pulse/min for 30 min, repeated daily) (red curve, square dots). Results are represented as mean ± SD and are the result of 3 biologically independent replicates. Statistics were performed with 2-way ANOVA multiple comparisons ***p < 0.001

We treated MSCs with 1 pulse/minute (approximately 17 mHz) for a duration of 30 min, repeated daily for 1 to 4 days. The choice of the duration of stimulation and the daily repetition was based on previous studies, in which, conversely, slowing down Ca^2+^ oscillations frequencies in MSCs under conditions of osteogenic induction with the use of DC electric fields for 30 min daily was shown to promote the osteogenic differentiation [17]. The number of cells in stimulated and non-stimulated control conditions were counted each day from day 1 to day 5, 24 h after the last stimulation (Fig. 7). We observed an increased number of cells, *i.e.*, an increased proliferation in the groups of MSCs treated with the µsPEFs, inducing Ca^2+^ oscillations at a frequency of 17 mHz. The difference in proliferation was visible from day 3, consistent with the long cycling times of MSCs (typically several days for human adipose derived MSCs) [32–35], and increased until the end of the experiment (at day 5). This could be the result of the first stimulation already inducing first differences in proliferation, which become increasingly visible with the days, or the result of the combined effects of each stimulation performed each day. To discriminate between these hypotheses, it would be necessary to evaluate the effect of the first stimulation only, until day 5. This was not performed in this study.

Importantly, these results show that imposing a frequency of Ca^2+^ oscillations of 17 mHz, previously reported to correlate with strong RAS activation, effectively induces more proliferation compared to control, unstimulated conditions. This result is in accordance with the hypothesis emitted that increased Ca^2+^ oscillation frequencies would correlate with increased proliferation.

## Discussion

We observed that terminal differentiation of MSCs, in both adipogenic and osteogenic lineages, is associated with an arrest in Ca^2+^ oscillations. A common point in the commitment to terminal differentiation in adipogenesis and osteogenesis is the exit from the cell cycle, as both mature adipocytes and mature osteocytes are post-mitotic cells (i.e. do not have the ability to divide anymore) [18, 36]. This observation could be in line with the fact that Ca^2+^ oscillations have been reported to occur in MSCs in the G1 and S phases of the cell cycle [11].

Another common feature of both differentiations, observed before the arrest of the Ca^2+^ oscillations in the terminally differentiated cells, was a phase of decrease in the Ca^2+^ oscillations frequencies, as compared to the undifferentiated MSCs. This phase of decrease appeared before the accumulation of lipid vesicles within differentiating adipocytes in the case of adipogenic differentiation (Figs. 4 and 5), and prior to the apparition of the first traces of mineralized matrix in the osteogenic differentiation (Fig. 1). In parallel with this observation, it is interesting to mention that the cell fate is determined during the G1 phase of the cell cycle, where the Ca^2+^ oscillations occur. Depending on its progression through the G1 phase, a cell will either commit to terminal differentiation, commit to complete the ongoing cell cycle, enter into senescence or, even undergo cell death (note that the latter can also occur at other stages of the cell cycle) [37]. This is well established in the adipogenic differentiation of the MSCs, where it has been shown that there is a molecular competition between mitogenic and differentiation signals during the G1 phase of the cell cycle, leading to cell fate decisions [18]. Indeed, to commit to terminal differentiation into an adipocyte, the “pre-adipocyte” needs a critical threshold level of the protein PPARγ, the main key transcription factor in adipogenic differentiation, to be reached [18]. In response to differentiation signals, this critical threshold is almost exclusively reached in G1 phase. Thus, during the G1, if the competing mitogenic signals mediate the progression through the cell cycle beyond the restriction point before the critical level of PPARγ for terminal differentiation is reached, the cell commits to complete the ongoing cell cycle. The competition between mitogenic and differentiation signals then resumes in the G1 phase of the daughter cells [18]. Conversely, if the protein level of PPARγ increases sufficiently during the G1 phase to reach the critical threshold, the cell commits to terminal differentiation and simultaneously exits the cell-cycle. This cell cycle exit is mediated by PPARγ itself, which directly upregulates, the CDK inhibitor p21, as well as FKBPL which slows p21 degradation [18]. A direct implication of this competition model is that the longer the G1 phase, the greater the likelihood that PPARγ will reach its critical level to commit the cell to terminal adipogenic differentiation. This can be directly correlated to multiple reports of G1 phase lengthening as a common phenomenon observed in the cell cycles preceding terminal differentiation of cells [38, 39]. In this context, G1 lengthening has been associated to the activity of the CDKs-CDK inhibitors [40].

Very interestingly, it has been hypothesized that Ca^2+^ oscillations are not only a bystander of G1 phase but play a role in its progression [11] and thus would also play a role in the length of G1 phase. This hypothesis is strongly supported by the fact that treating MSCs with chemical inhibitors or activators of the actors involved in Ca^2+^ oscillations indeed has an effect on cell cycle progression and proliferation. For instance, treatment of MSCs with Xestospongin C (a potent inhibitor of the IP3 receptors) abolished Ca^2+^ oscillations, downregulated protein levels of cyclin E and CDK2 and upregulated protein levels of the CDK inhibitor p27. Conversely, treatment of MSCs with Adenophostin A (a potent IP3 receptor agonist) had the opposite effect on the cell cycle regulators [11]. Ultimately, the treatment of MSCs with various inhibitors of different actors involved in Ca^2+^ oscillations decreased cell proliferation compared to untreated control MSCs. On the contrary, treatment of MSCs with Adenophostin A or Thimerosal (which increases the sensitivity of IP3 receptors to IP3), had the opposite effect [11].

Most interestingly, Ca^2+^ oscillations were also reported to affect the activity of the small GTPase RAS and the subsequent Raf/MEK/ERK cascade (also known as the MAPK-ERK pathway) [21]. RAS has been identified as a driver of cell-cycle progression, especially at mid-G1 and G1/S transition by increasing cyclin D levels (via ERK or PI3K/Akt), which are required to progress up to and through the G1/S transition [41]. Taken together, this fits very well with studies showing that some cells depend on the presence of extracellular Ca^2+^ to progress through certain stages of the G1 phase, particularly in early G1 and close to the G1/S transition [42]. This may also be consistent with the fact that the levels of calmodulin, one of the major Ca^2+^ effectors, increase at the G1/S transition [43]. In more detail, RAS is active upon the binding of GTP and is deactivated by its intrinsic GTPase activity, hydrolyzing GTP to GDP. RAS has multiple regulators of its activity, RAS-GAPs (RAS specific GTPase-activating proteins) and RAS-GEFs (RAS specific guanine–nucleotide exchange factors), which enhance the low intrinsic GTPase activity of RAS and promote the exchange of GDP for GTP, respectively. Some of these regulators have been reported to have Ca^2+^ -sensitive activities (e.g., RASAL and CAPRI RAS-GAPs or RAS-GRF and RAS-GRP Ras-GEFs) [44, 45]. In this context, the activity of RAS has been shown to positively correlate with Ca^2+^ oscillations frequencies between 3 and 17 mHz (Ca^2+^ oscillation frequencies were modulated in the original study using a biochemical Ca^2+^ clamp technique, and thus higher frequencies could not be tested due to the experimental design)[21]. Ca^2+^ oscillation frequencies higher than 17 mHz might result in even greater RAS activation, but as mentioned, could not be assessed in the original study [21]. As a potential insight into the answer to this open question, it was at least shown that MSCs exposed to an imposed 33 mHz Ca^2+^ oscillation frequency for 30 min display a higher expression of EGR1 (Early Growth Response 1), CCND1 (Cyclin D1) and CCNE1 (Cyclin E1) compared to untreated control cells [46]. In addition, a slightly higher proportion of cells in the S and G2/M phases of the cell cycle started to be visible 24 h after the treatment compared to untreated control MSCs [46]. Remarkably, the frequency range of Ca^2+^ oscillations reported in [21] is almost exactly the range of the Ca^2+^ oscillations frequencies exhibited by MSCs in our study during proliferation and differentiation (Figs. 2 and Fig. 6) [31, 42, 43].

In the undifferentiated state or at the beginning of the osteogenic differentiation, when cells are still actively dividing [31] and that the ERK-MAPK pathway is known to be strongly activated [47], the Ca^2+^ oscillation frequencies that we observed are in the range that has been reported to correlate with important RAS activation, and subsequently, would correlate with progression through G1 phase and the G1/S transition. Later in the differentiation processes (in both the osteogenic and the adipogenic pathways), the observed decrease in the frequency of Ca^2+^ oscillations might correlate with lower RAS activation, and subsequently, with lengthened G1 phases, preparing the differentiating cells to commit to terminal differentiation. It is important to note that, in order to fully validate our hypothesis, it would have been important to experimentally verify the change in the RAS-GDP/RAS-GTP balance. However, this is highly challenging, as there are no antibodies capable of detecting this subtle difference, rendering techniques like Western blot and PCR insufficient. The RAS-GDP/RAS-GTP balance fluctuates throughout the cell cycle, regardless of *ras* gene transcription levels. While the balance between ERK and phosphorylated ERK can be measured as the endpoint of the MAP kinase pathway, RAS is not the only upstream activator in the cascade leading to ERK phosphorylation. As a result, we were unable to provide direct evidence to support this hypothesis, though it remains an appealing prospect.

Taken together, all of the above elements suggest that if the aim is to influence the cell fate towards proliferation of (undifferentiated) MSCs, increasing the Ca^2+^ oscillation frequency seems to be an achievable and interesting strategy. Conversely, if the aim is to influence the MSCs cell fate towards differentiation, decreasing their Ca^2+^ oscillation frequency under differentiating conditions (*i.e.,* in presence of differentiating factors) could be an appropriate approach. Actually, decreasing the Ca^2+^ oscillation frequencies in MSCs undergoing osteodifferentiation with the application of direct current electric fields, had previously been shown to facilitate the differentiation process [17].

With respect to our hypotheses, our results confirm that MSCs treated with µsPEFs to increase their Ca^2+^ oscillation frequencies to 17 mHz for 30 min per day for 1 to 4 days exhibit greater proliferation compared to untreated control MSCs. The effect of the treatment on cell proliferation became visible from day 3 of the experiment, in line with cycling times of several days, and further increased until day 5 (end of the experiment), indicating that no major damage was caused to the cells by the daily repetition of the µsPEF treatment.

## Conclusion

We observed that a common feature in the later stages of MSCs differentiation was a progressive decrease in Ca^2+^ oscillation frequencies, followed by a complete cessation of oscillations as the differentiation reached its term. The arrest of Ca^2 +^ oscillations appears to be associated with terminal differentiation and cell cycle exit. Based on the current knowledge available in literature and our own observations, a relationship seems to exist between Ca^2+^ oscillation frequencies, progression of cells through the G1 phase and ultimately the commitment to complete the cell cycle or the commitment to terminal differentiation.

This led us to hypothesize that increasing the Ca^2+^ oscillation frequencies compared to those exhibited by MSCs in classical culture conditions could favor cell proliferation. We experimentally confirmed this hypothesis by controlling the frequency of the Ca^2+^ oscillations using µsPEFs. Thus, we show that it is possible to control the fate of MSCs to, at the least, maintain, and even increase their growth rate. These results support future work on the modulation of MSCs proliferation or differentiation by means of a very mild membrane electropermeabilization to Ca^2+^ ions, allowing the manipulation of MSCs Ca^2+^ oscillations. This is a powerful strategy that would allow the regulation of the cell fate by a well controllable physical method.

Among the new perspectives offered by this control of the MSCs fate, the possibility of accelerating the proliferation of the MSCs is of great interest. Indeed, MSCs grow slowly, and for both experimental and therapeutic processes, it can take a long time to reach the required number of cells. This obstacle may be overcome by the delivery of µsPEFs as shown here, coupled with the use of appropriate devices to repeatedly expose the cells to the electric pulses under sterile conditions. Such an application could be performed both in vitro as well as in vivo, in specifically designed electrified bioimplants [48].

## Data Availability

Data are available upon request to the authors.

## Abbreviations

MSC: Mesenchymal stem cells
μsPEFs: Microsecond pulsed electric fields
DC: Direct current
ECM: Extracellular matrix
ALP: Alkaline phosphatase
P2Y1: Metabotropic purinergic G-protein associated receptor
PLC-β: Phospholipase C, beta subtype
TKR: Tyrosine kinase receptor
IP3: Inositol trisphosphate
ERK: Extracellular signal-regulated kinases
PI3K: Phosphatidyl inositol 3-kinases
Akt: Kinase from the thymoma-producing virus in Ak mice
GTP: Guanosine triphosphate
RAS: GTPase named after rat sarcoma
RAS-GAPs: RAS specific GTPase-activating proteins
PPARγ: Peroxisome proliferator-activated receptor gamma
CDK: Cyclin-dependent kinases
ANOVA: Analysis of variance
pNPP: Para-nitrophenyl phosphate
MAPK: Mitogen-activated protein kinases
